# Drug Investigation to Dampen the Comorbidity of Rheumatoid Arthritis and Osteoporosis via Molecular Docking Test

**DOI:** 10.3390/cimb44030069

**Published:** 2022-02-23

**Authors:** Ki-Kwang Oh, Md. Adnan, Dong-Ha Cho

**Affiliations:** Department of Bio-Health Convergence, College of Biomedical Science, Kangwon National University, Chuncheon 24341, Korea; nivirna07@kangwon.ac.kr (K.-K.O.); mdadnan1991.pharma@gmail.com (M.A.)

**Keywords:** rheumatoid arthritis, osteoporosis, comorbidity, interleukin 6, MD2-TLR4-IN-1

## Abstract

At present, most rheumatoid arthritis (RA) patients are at risk of osteoporosis (OP), which is increased by 1.5 times compared to non-RA individuals. Hence, we investigated overlapping targets related directly to the occurrence and development of RA and OP through public databases (DisGeNET, and OMIM) and literature. A total of 678 overlapping targets were considered as comorbid factors, and 604 out of 678 were correlated with one another. Interleukin 6 (IL-6), with the highest degree of value in terms of protein–protein interaction (PPI), was considered to be a core target against comorbidity. We identified 31 existing small molecules (< 1000 g/mol) as IL-6 inhibitors, and 19 ligands were selected by the 3 primary criteria (Lipinski’s rule, TPSA, and binding energy). We postulated that MD2-TLR4-IN-1 (PubChem ID: 138454798), as confirmed by the three criteria, was the key ligand to alleviate comorbidity between RA and OP. In conclusion, we described a promising active ligand (MD2-TLR4-IN-1), and a potential target (IL-6) against comorbidity of RA and OP, providing scientific evidence for a further clinical trial.

## 1. Introduction

Rheumatoid arthritis (RA) is a chronic systemic inflammatory disease that mainly causes severe pain and is associated with physical unfitness, diverse comorbidities, and diminished quality of life [1,2]. The main symptoms of RA are musculoskeletal pain, swelling, and stiffness of affected joints, linked deeply to synovial inflammation [3,4]. RA can present at all ages, and around 1% of this population suffers intractable pain, which entails enormous emotional stress and economic burden for the individual, and even for society [5]. Inflammation is the main driving factor that causes joint impairment, disorder, and unexpected comorbidity in RA patients, and anti-inflammation is the most significant therapeutic strategy [6]. At present, there are seven antibody drugs (biologics) for the treatment of RA: infliximab, adalimumab, etanercept, golimumab, tocilizumab, certolizumab, and abatacept [7]. In particular, disease-modifying anti-rheumatic drugs (DMARDs) are targeted against RA inflammation, limited to connective tissue damage [8]. Moreover, biological DMARDs are ineffective for improving bone density involved in the development of OP [9].

Osteoporosis (OP) is a severe health condition that weakens bones, making them fragile and more easily destroyed [10]. OP symptoms include back pain, loss of height, bone fractures, and change in posture [11]. Similarly, OP can also occur at all ages; mainly, primary osteoporosis develops ~10–15 years after menopause in women, and in elderly men between 75 and 80 years old [12]. In 2017, the International Osteoporosis Foundation announced that around 33% of women over 50 years old and 20% of men would experience OP in their lifespan [13]. Most recently, an emerging significant factor in OP was found to be inflammation that occurs upon bone turnover [14]. Thus, blockage of inflammation is a key clinical approach in OP patients [15]. Denosumab and odanacatib were used as biologics in the treatment of OP by enhancing bone mineral density [16,17]. Collectively, all biologics dampen the immune system, making it susceptible to common infections such as pneumonia, respiratory infections, urinary tract infections, and skin infections [18]. In addition, all antibody drugs are parenteral preparations for the patient, which have multiple risks, including hypersensitivity responses, risk of infection and emboli, and the absence of drug reversal [19,20,21]. Additionally, non-steroidal anti-inflammatory drugs (NSAIDs) are commonly used to relieve the pain related to RA and OP [22]. All NSAIDs are targeted to cyclooxygenase-1 (COX-1) and cyclooxygenase-2 (COX-2)—the two cyclooxygenase (COX) isoforms in tissues, which have different expression levels [23]. Moreover, the inhibition of COX interferes with bone formation, angiogenesis, and soft tissue regeneration, which obstructs the bone healing process [24]. This implies that NSAIDs function merely as an analgesic for a short period of time.

Most commonly, OP has been considered to be a classical comorbidity in RA [25]. According to one report, in a cohort of 47,000 RA patients, the risk of osteoporotic destruction increased by 1.5 times compared with non-RA individuals [26]. Both RA and OP represent chronic inflammatory responses against cytokines such as interleukin 1 (IL-1), interleukin 6 (IL-6), and interleukin 17 (IL-17) [27]. This implies that inhibition of interleukin(s) might be a crucial strategy to alleviate the liaisons between RA and OP. The application of drug repositioning analysis on a data-driven approach is the most efficient methodology to obtain promising compounds [28].

Furthermore, drug repositioning is the procedure of obtaining new therapies for already-existing drugs [29]. This process has great synergistic effects, diminishing the cost of new drug development as well as securing its safety [30]. Previously, the output of drug repositioning was mainly due to fortuitous findings of unexpected therapeutic effects identified after testing with a given agent [31]. However, at present, the development of computational methodologies from holistic perspectives provides us with critical hints to reevaluate the additional efficacy of existing drugs [32].

Thus, the aim of this work was to discover the hierarchical target by which to manage both RA and OP via the computational approach method, thereby unveiling the most significant small molecule (<1000 g/mol) against the comorbidity of the two diseases.

## 2. Hypothesis

The identified overlapping targets between RA and OP were used to construct protein–protein interaction (PPI). We hypothesized that a target with the highest degree of value would be the most promising therapeutic point [33], while a ligand with the lowest binding energy would be the most significant compound against the comorbidity of RA and OP.

## 3. Methods

### 3.1. Retrieval of RA or OP Targets and Identification of Overlapping Targets

The targets linked to the occurrence and development of RA and OP were retrieved from DisGeNET (https://www.disgenet.org/) (accessed on 24 July 2021), OMIM (https://www.omim.org/) (accessed on 26 July 2021), and previous literature. InteractiVenn was utilized to identify the overlapping targets between RA and OP.

### 3.2. PPI Network Analysis

The overlapping targets analyzed by STRING (https://string-db.org/) (accessed on 29 July 2021) had their PPI constructed via an R package. One target with the highest degree of value was obtained via PPI analysis; we considered it to be the most significant target to manage the comorbidity of RA and OP.

### 3.3. Collection of Ligands

Based on the target, we prepared for its known ligands on a small molecule screen (<1000 g/mol), which can facilitate its biological activity or modify a target [34]. The ligands were retrieved from the website of the chemical supplier Selleckchem (https://www.selleckchem.com/) (accessed on 2 August 2021), which had input them into PubChem (https://pubchem.ncbi.nlm.nih.gov/) (accessed on 2 August 2021) for identification in the SMILES (simplified molecular-input line-entry system) format.

### 3.4. The Screening of Ligands

The screening methodology of the selected ligands was based on three criteria (Lipinski’s rule, TPSA, and binding energy), which were filtered using SwissADME (http://www.swissadme.ch/) (accessed on 4 August 2021). The three detailed selective conditions were as follows: (1) Lipinski’s rule violation (≤1) [35], (2) TPSA (<140 Å^2^) [36], and (3) binding energy (<−6.0 kcal/mol) [37].

### 3.5. The Preparation of Ligands and a Target for MDT

The identified ligands were converted from .sdf on PubChem into .pdb format via PyMOL; finally, the ligands were converted into .pdbqt format using AutoDock. Likewise, the PDB ID of the target was identified via RCSB PDB (https://www.rcsb.org/) (accessed on 7 August 2021), which was selected as .pdb format and converted to .pdbqt format via AutoDock (http://autodock.scripps.edu/ 15 December 2021). The existing positive ligands were docked with a target on AutoDock 4 by setting up 4 energy ranges and 8 levels of exhaustiveness as default to obtain 10 different poses of ligand molecules [38]. The grid box size was set to 40 Å × 40 Å × 40 Å. The 2D binding interactions were utilized on LigPlot+ v.2.2 (https://www.ebi.ac.uk/thornton-srv/software/LigPlus/) (accessed on 8 August 2021). After the molecular docking test (MDT), a key ligand accepted by three criteria with the lowest binding energy (highest affinity) was selected to visualize the ligand–target complex in PyMOL.

### 3.6. The Prediction of Toxicological Properties of the Key Ligand in Silico

**Figure 1:**
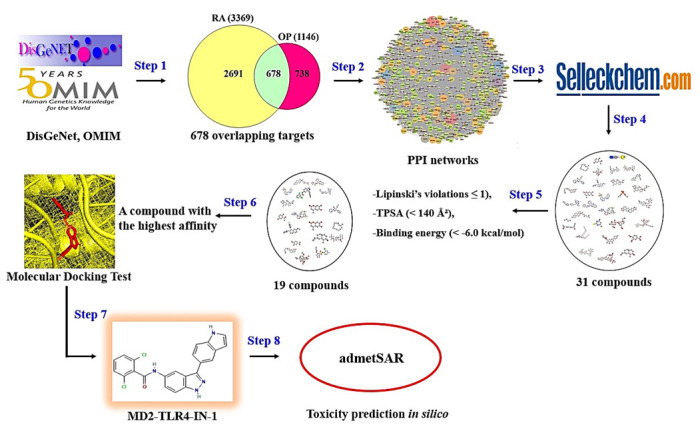
The workflow of this study.

Finally, we established the toxicological properties of the key ligand via the admetSAR web service tool (http://lmmd.ecust.edu.cn/admetsar1/predict/) (accessed on 10 August 2021) to develop new medication [39]. The workflow of this study is represented in Figure 1.

## 4. Results

**Figure 2:**
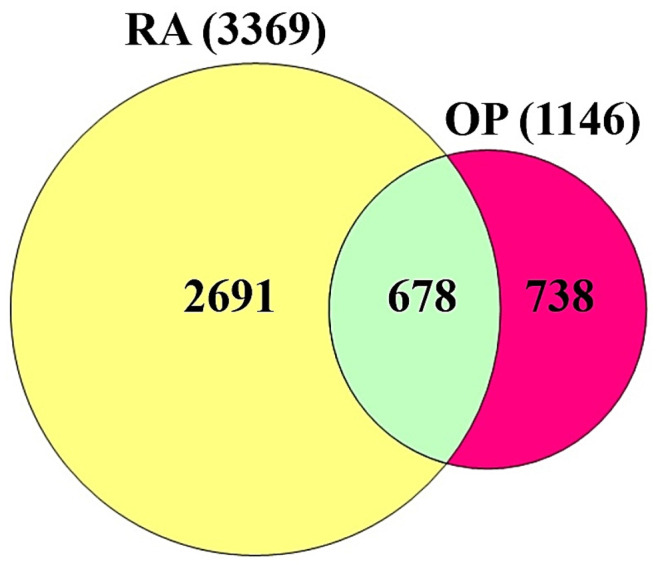
The overlapping targets between RA (3369 targets) and OP (1416 targets).

**Figure 3:**
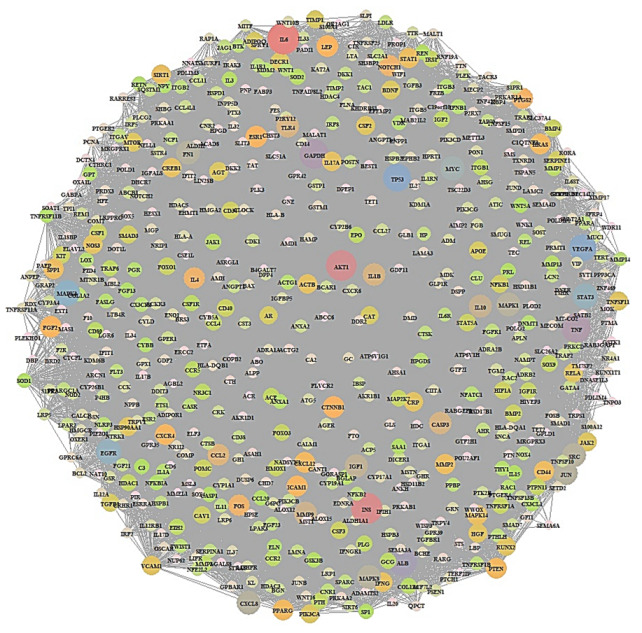
PPI network of 604 overlapping targets.

A total of 3369 targets associated with the occurrence and development of RA and a total of 1416 targets associated with OP were identified from DisGeNET (https://www.disgenet.org/ 15 December 2021), OMIM (https://www.omim.org/ 15 December 2021), and the literature (Supplementary Table S1). A total of 678 targets were overlapped between RA (3369 targets) and OP (1416 targets) (Figure 2) (Supplementary Table S2). Based on STRING analysis, 604 out of 678 targets were directly associated with comorbidity of RA and OP, suggesting 604 nodes and 16,705 edges (Figure 3); the 74 removed targets had no connectivity to the overlapping 678 targets. The nodes represented the total number of targets, while the edges stood for the number of relationships of each node. In PPI networks, IL-6 (432 degrees) had the greatest degree of value, and was considered a hierarchical target to manage the comorbidity of RA and OP (Table 1). The IL-6 (PDB ID: 4NI9) structure was revealed as two bound forms: apo-bound and receptor-bound [40,41]. The full length of IL-6 consisted of 212 amino acids linked to specific signal peptides with 29 amino acids, with a four-helix structure organized topologically [41,42]. In particular, 20 residues of the N-terminal did not form any secondary structure, and only the last 7 amino acids were identified as the crystal structure [43]. In contrast, the C–D loop of 10 residues (131–140 amino acids) was invisible in the crystal structure, and 17 (amino acids 44–60) out of 37 residues (amino acids 43–79) were unresolved in the crystal structure [43]. Then, IL-6 inhibitors of 31 small compounds (<1000 g/mol) to be retrieved from the Selleckchem website were screened by 3 criteria (Lipinski’s rule, TPSA, and binding energy) (Table 2); 19 out of 31 compounds were sorted by the three criteria. The MDT profiling of 31 known IL-6 inhibitors is given in Table 3.

Particularly, forsythoside B (PubChem ID: 23928102) and pectolinarin (PubChem ID: 168849), with a higher affinity for IL-6 than MD2-TLR4-IN-1 (PubChem ID: 138454798), were not accepted by the Lipinski’s rule (Lipinski’s violations ≤ 1) and TPSA (<140 Å^2^) criteria.

**Figure 4:**
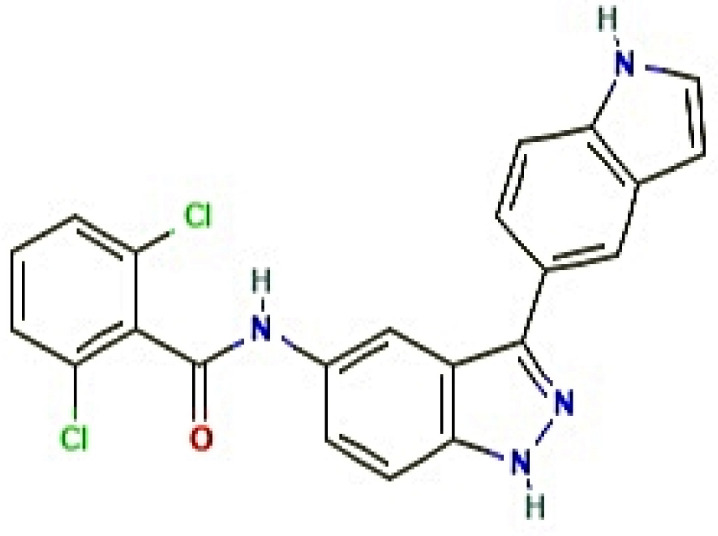
2D structure of MD2-TLR4-IN-1 (PubChem ID: 138454798).

**Figure 5:**
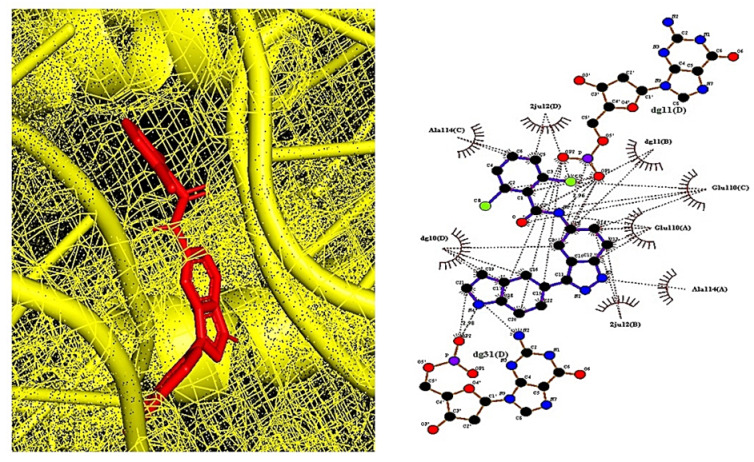
The molecular docking between IL-6 (PDB ID: 4NI9) and MD2-TLR4-IN-1 (PubChem ID: 138454798).

A total of 19 out of 31 compounds were accepted by Lipinski’s rule (Lipinski’s violations ≤ 1), TPSA (<140 Å^2^), and binding energy (<−6.0 kcal/mol), among which MD2-TLR4-IN-1 (PubChem ID: 138454798) (Figure 4), with the highest binding energy (−9.9 kcal/mol), was selected as the most important ligand to dampen comorbidity of RA and OP. MDT studies suggest that aprepitant (PubChem ID: 135413536), NE 52-QQ57 (PubChem ID: 68379135), and madecassic acid (PubChem ID: 73412) could be potential ligands with positive effects in alleviating comorbidity. The MDT of MD2-TLR4-IN-1 (PubChem ID: 138454798) on IL-6 (PDB ID: 4NI9) is displayed in Figure 5. The MDT results showed that the complex of IL-6 (PDB ID: 4NI9) with MD2-TLR4-IN-1 (PubChem ID: 138454798) had two hydrophobic interactions (Glu110 and Ala114), and there was no hydrogen bonding; this implies that hydrophobic interactions exert a strong binding effect on the complex of IL-6 (PDB ID: 4NI9) with MD2-TLR4-IN-1 (PubChem ID: 138454798). Furthermore, MD2-TLR4-IN-1 (PubChem ID: 138454798) on IL-6 had 4–22 donors and 3–7 acceptors; however, aprepitant (PubChem ID: 135413536), with the second highest affinity for IL-6, had 7–23 donors and 1–13 acceptors, while NE 52-QQ57 (PubChem ID: 6837915), with the third highest affinity for IL-6, had 11–24 donors and 2–7 acceptors. In parallel, these results shed light on the significance of the number of acceptors involved in target–ligand interactions. Finally, we demonstrated the toxicity of MD2-TLR4-IN-1 (PubChem ID: 138454798) via the admetSAR web-based tool. Our results showed that MD2-TLR4-IN-1 (PubChem ID: 138454798) had no Ames toxicity, carcinogenic properties, acute oral toxicity, or rat acute toxicity properties (Table 4).

## 5. Discussion

A total of 678 targets were involved in the occurrence and development of the liaison of comorbidity between RA and OP. IL-6, with the highest degree of value in PPI, was considered to be a core target to alleviate the level of pathological severity.

A report demonstrated that IL-6 inhibition prevents the progression of joint destruction in RA patients and interferes with bone resorption by blocking osteoclast formation [44]. IL-6 plays important roles in inflammatory processing—a continuation of autoimmunity via B-cell and T-helper 17 (Th17) differentiation [45]. Tocilizumab (TCZ), as a representative antagonist of IL-6, has been used to treat RA; however, its serious adverse side effect is that infectious disease related to C-reactive protein (CRP) cannot be recognized during TCZ treatment [46]; this implies that stealth infections without any specific signals can wreak havoc on patients’ condition.

Another report revealed that the expression level of IL-6 in OA patients is elevated noticeably, and that there is a considerable correlation between CRP and bone mineral density (BMD) [47]. Moreover, IL-6 expression levels were increased in the synovial fluid of RA patients, and a significant stimulator of bone resorption in OA patients [48]. Particularly, in an in vivo test, IL-6 aggravated the severity of osteoporosis, due to fewer osteoclasts and increased bone destruction [49]; this suggests that IL-6 plays a pivotal role in alleviating osteoporotic inflammation reactions.

Collectively, IL-6 inhibitors may be promising ligands to overcome comorbidity of RA and OP. Among the known IL-6 inhibitor ligands, MD2-TLR4-IN-1 (PubChem ID: 138454798), which is a derivative of indazole with a heterocyclic aromatic organic compound, was accepted by all three criteria (Lipinski’s rule, TPSA, and binding energy). The derivatives consist of a benzene ring and a pyrazole ring, which exert diverse biological activities, including antitumor, antibacterial, antifungal, antiarrhythmic, anti-HIV, and anti-inflammation activities [50,51]. A report demonstrated that derivatives of indazoles have potent anti-inflammatory efficacy, including anti-RA and anti-OP [52,53]. Thus, we suggest that MD2-TLR4-IN-1 (PubChem ID: 138454798) might be a potent ligand to alleviate the comorbidity of RA and OP.

## 6. Conclusions

To sum things up, the most significant targets—the key ligands for alleviation of the comorbidity of RA and OP—were IL-6 (PDB ID: 4NI9) and MD2-TLR4-IN-1 (PubChem ID: 138454798), respectively. This study gives us a hint at the value of imidazole derivatives to develop new medications against RA and OP.

## Figures and Tables

**Table 1:** Degrees of value of the top 20 targets from PPI.

No.	Target	Degrees of Value
1	IL-6	432
2	INS	317
3	AKT1	312
4	TNF	289
5	GAPDH	288
6	TP53	267
7	VEGFA	266
8	MAPK3	242
9	EGFR	237
10	STAT3	236
11	CXCL8	216
12	JUN	216
13	MAPK1	215
14	SRC	215
15	MMP9	215
16	IGF1	209
17	IL-10	206
18	CASP3	195
19	IL-1B	195
20	TLR4	194

**Table 2:** The physicochemical properties and classification of 31 compounds as IL-6 antagonists.

	Compounds	Lipinski Rules							
		MW	HBA	HBD	MLogP	Lipinski’s violations	Bioavailability Score	TPSA	Compound Classification
No.		<500	<10	≤5	≤4.15	≤1	>0.1	<140 Å^2^	
1	Forsythoside B	756.70	19	11	−3.93	3	0.17	304.21	Oligosaccharides
2	Pectolinarin	622.57	15	7	−3.03	3	0.17	227.20	Flavonoid-7-O-glycosides
3	MD2-TLR4-IN-1	421.28	2	3	4.01	0	0.55	73.57	Indazole
4	Aprepitant	534.43	12	2	4.05	1	0.55	83.24	Phenylmorpholines
5	Mulberroside A	568.52	14	10	−2.97	3	0.17	239.22	Stilbene glycosides
6	Homoplantaginin	462.40	11	6	−1.89	2	0.17	179.28	Flavonoid-7-O-glycosides
7	NE 52-QQ57	416.52	6	1	3.46	0	0.55	81.14	Pyrazolo[1,5-a]pyrimidines
8	Madecassic acid	504.70	6	5	3.33	1	0.55	118.22	Triterpenoids
9	GSK583	398.45	5	2	3.36	0	0.55	96.12	Aminoquinolines and derivatives
10	IQ 3	341.32	6	0	2	0	0.55	77.58	Quinoxalines
11	Methylprednisolone	374.47	5	3	1.52	0	0.55	94.83	21-Hydroxysteroids
12	Hydrocortisone hemisuccinate	462.53	8	3	1.29	0	0.55	138.20	Gluco/mineralocorticoids, progestogens, and derivatives
13	20(S)-Ginsenoside Rh1	638.87	9	7	1.77	2	0.17	160.07	Triterpene saponins
14	Stylopine	323.34	5	0	2.56	0	0.55	323.34	Protoberberine alkaloids and derivatives
15	Methylprednisolone Acetate	416.51	6	2	1.86	0	0.55	100.90	Gluco/mineralocorticoids, progestogens, and derivatives
16	Gardenoside	404.37	11	6	−2.62	2	0.11	175.37	Iridoid O-glycosides
17	4-Methylesculetin	192.17	4	2	0.76	0	0.55	70.67	6,7-Dihydroxycoumarins
18	Auraptene	298.38	3	0	3.51	0	0.55	39.44	Terpene lactones
19	AX-024 HCl	375.86	4	0	3.86	0	0.55	21.70	Neoflavenes
20	APX-115 free base	279.34	2	1	2.66	0	0.55	50.68	Pyrazolylpyridines
21	Resatorvid	361.82	5	1	2.44	0	0.55	80.85	Sulfanilides
22	Myrislignan	374.43	6	2	1.97	0	0.55	77.38	Lignans, neolignans, and related compounds
23	Muscone	238.41	1	0	3.92	0	0.55	17.07	Cyclic ketones
24	2′,5′-Dihydroxyacetophenone	152.15	3	2	0.51	0	0.55	57.53	Alkyl-phenylketones
25	α-Cyperone	218.33	1	0	3.46	0	0.55	17.07	Eudesmane, isoeudesmane, or cycloeudesmane sesquiterpenoids
26	Veratric acid	182.17	4	1	1.06	0	0.85	55.76	P-methoxybenzoic acids and derivatives
27	Triolein	885.43	6	0	9.49	2	0.17	78.90	Triacylglycerols
28	Methylthiouracil	142.18	1	2	−0.35	0	0.55	80.74	Pyrimidones
29	Falcarindiol	260.37	2	2	3.33	0	0.55	40.46	Long-chain fatty alcohols
30	Diethyl phosphate	154.10	4	1	−0.43	0	0.85	65.57	Dialkyl phosphates
31	Sodium thiocyanate	81.07	1	0	−1.01	0	0.55	23.79	Metal thiocyanates

**Table 3:** Binding energy of 31 known IL-6 inhibitors (<1000 g/mol).

				Grid Box		Hydrogen Bond Interactions	Hydrophobic Interactions
Protein	Ligand	PubChem ID	Binding Energy (kcal/mol)	Center	Dimension	Amino Acid Residue	Amino Acid Residue
IL6 (PDB ID: 4NI9)	Forsythoside B	23928102	−11.4	x = 11.213	size_x = 40	Asp34,Tyr31,Glu110	Gly35,Gln111,Ala114
				y = 33.474	size_y = 40		
				z = 11.162	size_z = 40		
	Pectolinarin	168849	−10.4	x = 11.213	size_z = 41	Asp34,Gln111	Ala38
				y = 33.474	size_z = 42		
				z = 11.162	size_z = 43		
	(*) MD2-TLR4-IN-1	138454798	−9.9	x = 11.213	size_z = 44	N/A	Glu110,Ala114
				y = 33.474	size_z = 45		
				z = 11.162	size_z = 46		
	(*) Aprepitant	135413536	−9.6	x = 11.213	size_z = 47	N/A	Tyr31,Asp34,Gly35
				y = 33.474	size_z = 48		Gln111
				z = 11.162	size_z = 49		
	Mulberroside A	6443484	−9.5	x = 11.213	size_z = 50	Glu110,Ser37,Asp34	Ala114,Gln111
				y = 33.474	size_z = 51	Tyr31	
				z = 11.162	size_z = 52		
	Homoplantaginin	5318083	−9.5	x = 11.213	size_z = 53	Asp34,Gln111	Ala38
				y = 33.474	size_z = 54		
				z = 11.162	size_z = 55		
	(*) NE 52-QQ57	68379135	−9.4	x = 11.213	size_z = 56	Ser37	Asp34,Tyr31,Ala114
				y = 33.474	size_z = 57		Gln111
				z = 11.162	size_z = 58		
	(*) Madecassic acid	73412	−9.4	x = 11.213	size_z = 59	Glu110	Ala114,Tyr31
				y = 33.474	size_z = 60		
				z = 11.162	size_z = 61		
	(*) GSK583	67469084	−9.0	x = 11.213	size_z = 62	N/A	Gln111,Ala38
				y = 33.474	size_z = 63		
				z = 11.162	size_z = 64		
	(*) IQ 3	777728	−9.0	x = 11.213	size_z = 65	N/A	Tyr31,Glu110
				y = 33.474	size_z = 66		
				z = 11.162	size_z = 67		
	(*) Methylprednisolone	6741	−9.0	x = 11.213	size_z = 68	N/A	Tyr31,Glu110
				y = 33.474	size_z = 69		
				z = 11.162	size_z = 70		
	(*) Hydrocortisone hemisuccinate	16623	−8.9	x = 11.213	size_z = 71	N/A	Glu110,Ala114,Gln111
				y = 33.474	size_z = 72		
				z = 11.162	size_z = 73		
	20(S)-Ginsenoside Rh1	12855920	−8.8	x = 11.213	size_z = 74	Gln111	Asp34,Tyr31
				y = 33.474	size_z = 75		
				z = 11.162	size_z = 76		
	Stylopine	6770	−8.8	x = 11.213	size_z = 77	N/A	Gln111,Ala114,Glu110
				y = 33.474	size_z = 78		
				z = 11.162	size_z = 79		
	(*) Methylprednisolone Acetate	5877	−8.6	x = 11.213	size_z = 80	N/A	Gln111,Tyr31,Ala114
				y = 33.474	size_z = 81		Glu110
				z = 11.162	size_z = 82		
	Gardenoside	24721095	−7.8	x = 11.213	size_z = 83	Tyr31,Asp34,Gln111	N/A
				y = 33.474	size_z = 84		
				z = 11.162	size_z = 85		
	(*) 4-Methylesculetin	5319502	−7.6	x = 11.213	size_z = 86	Arg24,Arg16	Pro18
				y = 33.474	size_z = 87		
				z = 11.162	size_z = 88		
	(*) Auraptene	1550607	−7.6	x = 11.213	size_z = 89	N/A	Asp34,Glu110,Ala114
				y = 33.474	size_z = 90		Tyr31
				z = 11.162	size_z = 91		
	(*) AX-024 HCl	129909862	−7.5	x = 11.213	size_z = 92	N/A	Gln111,Tyr31,Ala114
				y = 33.474	size_z = 93		
				z = 11.162	size_z = 94		
	(*) APX-115 free base	51036475	−7.2	x = 11.213	size_z = 95	Tyr31	Glu110,Gln111,Asp34
				y = 33.474	size_z = 96		
				z = 11.162	size_z = 97		
	(*) Resatorvid	11703255	−7.1	x = 11.213	size_z = 98	Tyr31,Gln111	Glu110,Asp34
				y = 33.474	size_z = 99		
				z = 11.162	size_z = 100		
	(*) Myrislignan	21636106	−7.1	x = 11.213	size_z = 101	Gln111	Tyr31,Gly35,Asp34
				y = 33.474	size_z = 102		
				z = 11.162	size_z = 103		
	(*) Muscone	10947	−6.7	x = 11.213	size_z = 104	N/A	N/A
				y = 33.474	size_z = 105		
				z = 11.162	size_z = 106		
	(*) 2′,5′-Dihydroxyacetophenone	10279	−6.5	x = 11.213	size_z = 107	N/A	Gln17,Pro18
				y = 33.474	size_z = 108		
				z = 11.162	size_z = 109		
	(*) α-Cyperone	6452086	−6.3	x = 11.213	size_z = 110	N/A	Gln111,Glu110
				y = 33.474	size_z = 111		
				z = 11.162	size_z = 112		
	(*) Veratric acid	7121	−6.1	x = 11.213	size_z = 113	Arg16	Gln17,Pro18
				y = 33.474	size_z = 114		
				z = 11.162	size_z = 115		
	Triolein	5497163	−5.5	x = 11.213	size_z = 116	N/A	N/A
				y = 33.474	size_z = 117		
				z = 11.162	size_z = 118		
	Methylthiouracil	667493	−5.4	x = 11.213	size_z = 119	N/A	Arg24
				y = 33.474	size_z = 120		
				z = 11.162	size_z = 121		
	Falcarindiol	5281148	−5.2	x = 11.213	size_z = 122	Glu110	Ala114,Tyr31,Gln111
				y = 33.474	size_z = 123		Glu110
				z = 11.162	size_z = 124		
	Diethyl phosphate	654	−4.9	x = 11.213	size_z = 125	N/A	Arg16,Gln17
				y = 33.474	size_z = 126		
				z = 11.162	size_z = 127		
	Sodium thiocyanate	516871	−2.6	x = 11.213	size_z = 128	N/A	Arg16,Gln17
				y = 33.474	size_z = 129		
				z = 11.162	size_z = 130		

(*): The indication of 19 compounds accepted by the three criteria: (1) Lipinski’s rule violation (≤1), (2) TPSA (<140 Å^2^), and (3) binding energy (<−6.0 kcal/mol).

**Table 4:** A prediction of toxicological propensity of MD2-TLR4-IN-1 (PubChem ID: 138454798).

Parameters	Compound
	MD2-TLR4-IN-1
Ames toxicity	NAT
Carcinogens	NC
Acute oral toxicity	Ⅲ
Rat acute toxicity	2.2347

NAT: Non-Ames toxic; NC: non-carcinogenic; Category II: 50 mg/kg > Lethal Dose 50% (LD50) < 500 mg/kg; Category III: 500 mg/kg > Lethal Dose 50% (LD50) < 5000 mg/kg; Category IV: Lethal Dose 50% (LD50) > 5000 mg/kg; Rat acute toxicity: the treatment of 2.2347 mol/kg in rats shows LD50 toxicity.

## Data Availability

All data generated or analyzed during this study are included in this published article (and its Supplementary Materials).

## Supplementary Materials

The following supporting information can be downloaded at https://www.mdpi.com/article/10.3390/cimb44030069/s1: Table S1: The 3377 rheumatoid arthritis (RA)-related targets, and 1426 osteoporosis (OP)-related targets; Table S2: The 678 overlapping targets.

## Abbreviations

BMD	Bone mineral density
COX	Cyclooxygenase
COX-1	Cyclooxygenase-1
COX-2	Cyclooxygenase-2
CRP	C-reactive protein
DMARDs	Disease-modifying anti-rheumatic drugs
IL-1	Interleukin 1
IL-6	Interleukin 6
IL-17	Interleukin 17
NSAIDs	Non-steroidal anti-inflammatory drugs
OP	Osteoporosis
PPI	Protein–protein interaction
RA	Rheumatoid arthritis
TCZ	Tocilizumab
TPSA	Topological polar surface area
